# Bringing Early Infant Male Circumcision Information Home to the Family: Demographic Characteristics and Perspectives of Clients in a Pilot Project in Tanzania

**DOI:** 10.9745/GHSP-D-15-00210

**Published:** 2016-07-02

**Authors:** Mbaraka Amuri, Georgina Msemo, Marya Plotkin, Alice Christensen, Dorica Boyee, Hally Mahler, Semakaleng Phafoli, Mustafa Njozi, Augustino Hellar, Erick Mlanga, Aisha Yansaneh, Emmanuel Njeuhmeli, Jackson Lija

**Affiliations:** a Jhpiego and AIDSFree, Dar es Salaam, Tanzania; b Ministry of Health, Community Development, Gender, Elderly and Children, Dar es Salaam, Tanzania; c Jhpiego and AIDSFree, Baltimore, MD, USA; d Jhpiego, Maseru, Lesotho; e U.S. Agency for International Development (USAID), Dar es Salaam, Tanzania; f USAID, Washington, DC, USA

## Abstract

Iringa region of Tanzania has had great success reaching targets for voluntary medical male circumcision (VMMC). Looking to sustain high coverage of male circumcision, the government introduced a pilot project to offer early infant male circumcision (EIMC) in Iringa in 2013. From April 2013 to December 2014, a total of 2,084 male infants were circumcised in 8 health facilities in the region, representing 16.4% of all male infants born in those facilities. Most circumcisions took place 7 days or more after birth. The procedure proved safe, with only 3 mild and 3 moderate adverse events (0.4% overall adverse event rate). Overall, 93% of infants were brought back for a second-day visit and 71% for a seventh-day visit. These percentages varied significantly by urban and rural residence (97.4% urban versus 84.6% rural for day 2 visit; 82.2% urban versus 49.9% rural for day 7 visit). Mothers were more likely than fathers to have received information about EIMC. However, fathers tended to be key decision makers regarding circumcision of their sons. This suggests the importance of addressing fathers with behavioral change communication about EIMC. Successes in scaling up VMMC services in Iringa did not translate into immediate acceptability of EIMC. EIMC programs will require targeted investments in demand creation to expand and thrive in traditionally non-circumcising settings such as Iringa.

## Abstract (precis)

During a pilot project in Tanzania’s Iringa region, more than 2,000 male infants were circumcised in less than 2 years in 8 facilities, representing 16.4% of all male births in those facilities. The age of the infant at circumcision and the time of return for follow-up visits varied significantly between urban and rural dwellers. Early infant male circumcision (EIMC) outreach activities and use of health outposts for follow-up visits should be explored to overcome these geographic barriers. EIMC programs will also require targeted investments in demand creation, especially among fathers, to expand and thrive in traditionally non-circumcising settings such as Iringa.

## INTRODUCTION

Three randomized controlled trials showed unequivocally that voluntary medical male circumcision (VMMC) reduces female-to-male HIV transmission by approximately 60%.^1^^–^^3^ In March 2007, the World Health Organization (WHO) and the Joint United Nations Programme on HIV/AIDS (UNAIDS) issued guidance urging countries with high HIV prevalence, low male circumcision rates, and a generalized HIV epidemic to incorporate VMMC into their comprehensive HIV prevention programs.^4^

Although Tanzania had an overall circumcision prevalence of 66.8% at the start of the VMMC program in 2009,^5^ Iringa region had a lower circumcision prevalence (29% of male adults) and at the time was Tanzania’s most HIV-affected region, with an adult HIV prevalence of 15.7%.^5^ Therefore, the Tanzanian Ministry of Health and Social Welfare (MOHSW) selected Iringa as a priority region for VMMC scale-up. The MOHSW, with support from the President’s Emergency Plan for AIDS Relief (PEPFAR) through the United States Agency for International Development (USAID), initiated VMMC services in Iringa region in 2009. As of December 2014, more than 272,740 adolescents (ages 10+ years) and adults had been circumcised through the VMMC program in Iringa, meeting the 2010 regional targets for VMMC of 264,990 circumcisions. Correspondingly, the adult male circumcision prevalence in Iringa increased from 29% to more than 60% between 2007–2008 and 2011–2012.^5^ Sustaining VMMC coverage over the long term will require circumcising adolescents, infants, or both. Modeling has projected that rolling out early infant male circumcision (EIMC) from 2013 through 2050 in Tanzania could avert 2% of all HIV infections and decrease overall HIV-related costs by 7%.^6^

Rolling out EIMC from 2013 through 2050 in Tanzania could avert 2% of all HIV infections.

EIMC, which has been recommended by WHO and UNAIDS as an HIV prevention strategy,^7^ entails surgical removal of the foreskin of male infants in a facility setting. EIMC is performed traditionally in many African countries, including Tanzania, with circumcision in infancy often associated with being Muslim. While adult VMMC is often described as a “catch-up” strategy, EIMC is seen to be more of a long-term or sustainable approach; as more infants are circumcised, fewer adolescents and adults will need circumcision in the future. When compared with VMMC, EIMC is easier to perform (does not require sutures), requires less time, and heals more quickly.^8^ WHO recommends that EIMC be performed within 24 hours to 60 days after birth.^8^

A systematic review of the literature on adverse events (AEs) associated with male circumcision found that EIMC was associated with the lowest rate of AEs when compared with child and adult male circumcision; EIMC had a median frequency of any complication (including moderate AEs) of 1.5%.^9^ It has been assumed that there may be fewer barriers to the service for infants than for boys or men. For example, the abstinence period following circumcision, which has been shown to be a major barrier to men seeking VMMC services, is not a barrier to EIMC.^10^ Generally low levels of implementation and evaluation to date stimulate additional research to seek solutions to challenges in scaling up EIMC.^11^

**FIGURE 1:**
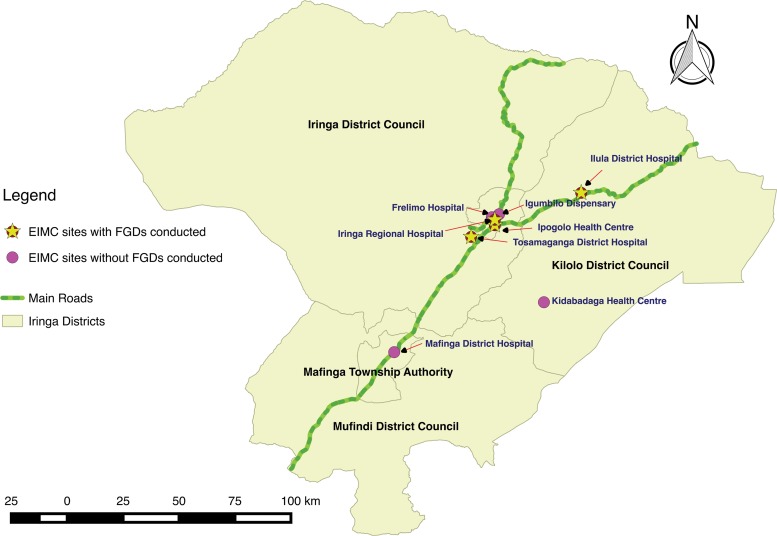
Facilities in the EIMC Pilot Project, Iringa Region, Tanzania Abbreviations: EIMC, early infant male circumcision; FGD, focus group discussion.

Beginning in April 2013, EIMC services were pilot-tested in 4 health facilities in Iringa region of Tanzania. Four more sites were added in April 2014 (Figure 1). From the pilot project’s inception through December 2014, more than 2,000 EIMCs were performed.

In the pilot program, EIMC services were offered in the outpatient reproductive and child health (RCH) services and conducted by health care providers who routinely provide services in the RCH or maternity ward. Counseling on EIMC, using standardized information from job aids, took place during antenatal care (ANC), maternity care, and/or postpartum care as well as during well-baby visits. Parents who heard of EIMC through counseling, community workers, or radio advertisements requested to have their child circumcised and were given appointments. Before the circumcision, parents/guardians were individually counseled on the risks and benefits of EIMC, offered HIV testing and counseling for themselves and their infants, informed about postoperative care, and asked to sign a written consent statement authorizing the procedure. The services were offered 3 to 5 days a week, depending on the facility. Parents were instructed to bring their infants back for follow-up on day 2 and day 7 after the circumcision procedure.

Iringa region was selected to host the EIMC pilot program as it was near to achieving the adult VMMC targets. It was unclear to what extent the gains in adult VMMC would translate into support for infant male circumcision; historically, the non-Muslim communities of Iringa region do not circumcise male infants. This paper presents findings from a quantitative and qualitative study conducted among users of the EIMC services and parents and guardians in the communities surrounding the EIMC pilot facilities. Demographic characteristics of clients in the EIMC pilot program are described, including the timing of circumcision and attendance at follow-up visits, as well as key qualitative findings associated with EIMC decision making. This paper aims to provide information to help guide rollout of EIMC services in Tanzania and similar settings in sub-Saharan Africa.

## METHODS

We conducted a cross-sectional study, using qualitative and quantitative methods, from May through August 2014. We conducted qualitative interviews with parents and guardians of EIMC clients in the 4 original pilot facilities as well as a review of EIMC patient files. The MOHSW and the regional authorities participated in selecting the EIMC pilot facilities, based on facility assessments that considered the number of deliveries in facilities (preference was given to higher volume), available human resources, availability of space, and capability for instrument processing/sterilization.

### Qualitative Component

The qualitative component of the study took place between May and August 2014 in the original 4 pilot facilities: Ipogolo Health Centre, Iringa Regional Referral Hospital, Tosamaganga Hospital, and Ilula Lutheran Hospital.

Study participants were parents/guardians receiving antenatal care service, parents/guardians of infants circumcised in the EIMC facilities, and parents/guardians who brought their male infants to RCH services and received information about EIMC but chose not to have the infants circumcised within 60 days of birth. We invited study participants to attend focus group discussions (FGDs). We conducted separate FGDs with the parents/guardians who had the infants circumcised through EIMC services (EIMC acceptors) and with parents/guardians who decided not to circumcise the infants through EIMC services (EIMC non-acceptors).

Parents and guardians who were recruited for the study during ANC services were followed over time until 2 months after the birth of the infants; they were then grouped in the FGDs as acceptors or non-acceptors, depending on their decision to circumcise the infants. All study participants were 18 years old or older, lived within a study health facility catchment area, and consented to participate in the research. We held 24 FGDs, with a range of 4 to 10 participants per FGD and an average of 7 participants. In all, 165 people took part in FGDs (Table 1).

**TABLE 1:** Overview of EIMC Study Participants

EIMC Acceptors				EIMC Non-Acceptors			
	No. of FGDs	Total No. of Participants	Range per FGD	No. of FGDs	Total No. of Participants	Range per FGD	Total No. of Participants
Men	4	28	4–10	4	24	4–7	**52**
Women	8	63	5–10	8	50	4–10	**113**
**Total**	**12**	**91**	**4–10**	**12**	**74**	**4–10**	**165**

Abbreviations: EIMC, early infant male circumcision; FGD, focus group discussion.

EIMC acceptors were recruited for FGDs on the day that the infants were circumcised and given an FGD appointment for a date within 2 weeks. They received reminders about the FGD 7 days after the circumcision, when parents or guardians brought the infants for the EIMC day 7 follow-up visit. FGD appointments for EIMC non-acceptors were scheduled within 2 weeks of recruitment.

Experienced and trained researchers conducted the FGDs in Kiswahili, using separate structured FGD guides for EIMC acceptors and for EIMC non-acceptors. These guides used open-ended questions to explore perspectives on decision making about EIMC.

The training for all study staff included research ethics and informed consent. All discussions were audio recorded, transcribed in Kiswahili, and then translated into English. Codes were grouped into categories, and emerging themes were then identified iteratively following the principles of grounded theory.^12^^,^^13^ A team of researchers (not the facilitators of the FGDs) reviewed and reached consensus on the themes. They based their analysis on theme saturation—once no new issues emerged, the description of the theme was considered exhaustive.

### Quantitative Component

The quantitative component of the study involved reviewing the records of EIMC clients in all 8 pilot facilities (the original 4 facilities and 4 additional facilities: Frelimo Hospital, Mafinga District Hospital, Kidabaga Health Centre, and Igumbilo Dispensary). During the pilot project, providers filled in MOHSW-approved EIMC client record forms, which were kept at each facility. These records were stripped of identifiers and entered into a program monitoring database. The de-identified client records were pulled from the program database and imported for analysis into Stata statistical software (StataCorp. 2013. *Stata Statistical Software: Release 13*. College Station, TX: StataCorp LP). Variables of interest included location of birth, location of circumcision, number of days of age at circumcision, follow-up appointments at 2 days and 7 days, rural versus urban residence of the mother, HIV status of the mother, and adverse events occurring during or after circumcision.

Using Stata, we conducted both descriptive analysis and bivariate logistic regression. Descriptive analysis was used to determine mean age of the infants and standard deviation. We used frequencies to describe key characteristics of EIMC clients, with comparisons among facilities, by urban and rural residence, and by infant’s age at circumcision. For comparison of 2 proportions, we used chi-square (χ^2^) tests for statistical significance of the observed difference. We conducted bivariate logistic regression to determine the odds of returning to the clinic for the infant’s follow-up care among mothers residing in urban locations and those in rural locations. The outcome variable was whether the infant was brought back for the follow-up visit, and the independent/predictive variable was rural/urban residence.

In this paper, age at circumcision is presented in age strata, which we constructed based on Tanzania’s clinical management protocols for the postpartum period, the immunization schedule for young infants, and the WHO recommendation to provide EIMC within 60 days of birth. In Tanzania, the MOHSW recommends return visits for routine postpartum care and immunization at 7, 28, and 42 days. Age at circumcision is presented in reference to those milestones.

### Ethical Considerations

Informed consent was required for all study participants, in addition to the nationally mandated consent required of parents or guardians before the infant underwent circumcision. The institutional review boards of the Johns Hopkins Bloomberg School of Public Health (IRB 00005145) and the Tanzania National Institute of Medical Research (NIMR/HQ/R.8a/Vol.IX/1684) conducted ethical oversight of the study with support from the Iringa regional medical authorities.

## RESULTS

### Demographic Characteristics of Infants in the EIMC Pilot Project

Table 2 presents characteristics of 2,084 infants circumcised during the pilot project, from April 2013 through December 2014. Of these infants, 1,398 had been delivered in one of the 8 participating facilities, 555 in other facilities that do not currently offer EIMC services, and 131 at home. During the pilot period, 12,678 male infants in total were born in the 8 pilot facilities; 16.4% of these were circumcised through the EIMC pilot program. Of the circumcised infants, 7.6% were born to HIV-infected mothers. The median age of the infants at circumcision was 22 days (range, 1–78 days), with variation by health facility, place of residence, and location of the baby’s delivery. Two-thirds (67.1%) of EIMCs were performed in the facility where the mother delivered her child, 26.6% in another facility, and a relatively small proportion (6.3%) at home. Almost two-thirds (64.2%) of EIMCs occurred among infants residing in urban areas, compared with 34.9% from rural areas. Overall, 93% of infants were brought back for the day 2 visit and 71% for the day 7 visit. This varied significantly by urban and rural residence—97.4% urban versus 84.6% rural for day 2 visit and 82.2% urban versus 49.9% rural for day 7 visit (*P*<.001) (Table 3).

Of the 12,678 male infants born in the 8 facilities, 16.4% were circumcised through the pilot program.

**TABLE 2:** Characteristics of Clients in the Iringa EIMC Pilot Project, April 2013–December 2014

	Iringa Regional Referral Hospital	Ipogolo Health Centre	Tosamaganga District Hospital	Ilula Lutheran Hospital	Frelimo Hospital	Igumbilo Dispensary	Mafinga District Hospital	Kidabaga Health Centre	Total
**No. of male infants born during pilot project**	**5,175**	**851**	**2,203**	**1,674**	**174**	**54**	**2,442**	**105**	**12,678**
EIMC uptake among male infants, No. (%)	483 (9.3)	268 (31.5)	303 (13.7)	263 (15.7)	66 (36.9)	36 (66.7)	86 (3.5)	24 (22.9)	2084 (16.4)
Age at circumcision, days, median (range)	32 (1–74)	27 (1–72)	4 (1–78)	14 (1–78)	28 (1–60)	30 (1–71)	14 (1–73)	3 (1–34)	22 (1–78)
**No. of EIMC performed**	**528**	**503**	**326**	**297**	**179**	**125**	**99**	**27**	**2,084**
EIMCs by mother’s place of delivery, No. (%)									
This facility	450 (85.2)	216 (42.9)	297 (91.1)	252 (84.9)	54 (30.2)	20 (16.0)	85 (85.9)	24 (88.9)	1,398 (67.1)
Another facility not offering EIMC services	45 (8.5)	235 (46.7)	23 (7.1)	34 (11.4)	113 (63.1)	89 (71.2)	13 (13.1)	3 (11.1)	555 (26.6)
Home	33 (6.3)	52 (10.3)	6 (1.8)	11 (3.7)	12 (6.7)	16 (12.8)	1 (1.0)	0 (0.0)	131 (6.3)
EIMCs by place of residence,^a^ No. (%)									
Urban	477 (90.3)	409 (81.3)	22 (6.7)	169 (56.9)	172 (96.1)	47 (37.6)	43 (43.3)	0 (0.0)	1,339 (64.2)
Rural	42 (8.0)	93 (18.5)	303 (92.9)	122 (41.1)	6 (3.3)	78 (62.4)	56 (56.6)	27 (100.0)	727 (34.9)
Infants brought for day 2 follow-up,^a^ No. (%)	510 (98.3)	495 (98.6)	210 (64.6)	289 (99.3)	172 (96.6)	125 (100)	91 (91.9)	27 (100.0)	1,919 (92.9)
Infants brought for day 7 follow-up,^a^ No. (%)	455 (87.7)	424 (84.5)	34 (10.5)	214 (73.5)	148 (83.2)	114 (91.2)	59 (59.6)	16 (59.3)	1,464 (70.9)
Circumcised infants with HIV+ mothers, No. (% of all circumcised infants)	34 (6.4)	27 (5.4)	28 (8.6)	34 (11.4)	14 (7.8)	8 (6.4)	13 (13.1)	1 (3.7)	159 (7.6)

Abbreviation: EIMC, early infant male circumcision.

a 18 mothers reported residence outside of the region and were excluded from this analysis.

**TABLE 3:** Place of Delivery and Follow-up Attendance by Urban or Rural Residence of Mother, Iringa EIMC Pilot Project, April 2013–December 2014

Indicator	Urban-Dwelling Mothers (N = 1,339)	Rural-Dwelling Mothers (N = 727)	*P* Value	Total (N = 2,066)^a^
	% (95% CI)	% (95% CI)		% (95% CI)
Mother’s place of delivery			<.001	
This facility	63.4 (60.9, 65.9)	73.7 (70.4, 76.8)		67.0 (65.0, 69.1)
Another facility not offering EIMC services	29.6 (27.2, 32.1)	21.3 (18.5, 24.4)		26.7 (24.8, 28.6)
Home	7.0 (5.8, 8.5)	5.0 (3.6, 6.8)		6.3 (5.3, 7.4)
Returned for day 2 follow-up visit	97.4 (96.4, 98.1)	84.6 (81.8, 87.0)	<.001	92.9 (91.7, 93.9)
Returned for day 7 follow-up visit	82.2 (80.1, 84.2)	49.9 (46.3, 53.6)	<.001	70.9 (68.9, 72.8)
Attended with the infant for circumcision			<.001	
Mother/female guardian	82.3 (80.2, 84.3)	68.8 (65.3, 72.0)		77.5 (75.7, 79.2)
Both parents	17.7 (15.7, 19.8)	31.2 (27.9, 34.7)		22.5 (20.7, 24.3)

Abbreviations: CI, confidence interval; EIMC, early infant male circumcision.

a 18 mothers reported residence outside of the region and were excluded from this analysis.

As Table 4 shows, the majority of infants (73.1%) were circumcised between 8 and 60 days of age. Approximately one-quarter (26.2%) of the infants were circumcised within 7 days of birth, ranging from 13.1% in health centers to 22.4% in dispensaries. Half of the infants (52.5%) were circumcised in the 8- to 42-day range, which coincides with the second and third scheduled immunization visits (28 days and 42 days) in Tanzania.

**TABLE 4:** Age at Circumcision by Level of Health Facility in the Iringa EIMC Pilot Project, April 2013–December 2014

	Age at Circumcision					
Facility Level (No. of EIMCs performed)	≤2 Days % (95% CI)	3–7 Days % (95% CI)	8–28 Days % (95% CI)	29–42 Days % (95% CI)	43–60 Days % (95% CI)	61–78 Days % (95% CI)
Hospitals (1,429)	12.0 (10.4, 13.8)	19.3 (17.3, 21.4)	29.2 (26.9, 31.7)	18.1 (16.1, 20.1)	20.6 (18.5, 22.7)	0.8 (0.4, 1.4)
Health centers (530)	3.8 (2.4, 5.8)	9.3 (7.1, 12.0)	41.9 (37.7, 46.1)	22.5 (19.1, 26.2)	21.7 (18.4, 25.4)	0.9 (0.4, 2.2)
Dispensaries (125)	12.8 (7.9, 20.0)	9.6 (5.5, 16.2)	22.4 (15.8, 30.6)	38.4 (30.2, 47.3)	16.0 (10.5, 23.6)	0.8 (0.1, 5.6)
Total (2,084)	9.9 (8.8, 11.3)	16.2 (14.6, 17.8)	32.1 (30.1, 34.1)	20.4 (18.7, 22.2)	20.6 (18.9, 22.4)	0.8 (0.5, 13.1)

Abbreviations: CI, confidence interval; EIMC, early infant male circumcision.

**FIGURE 2:**
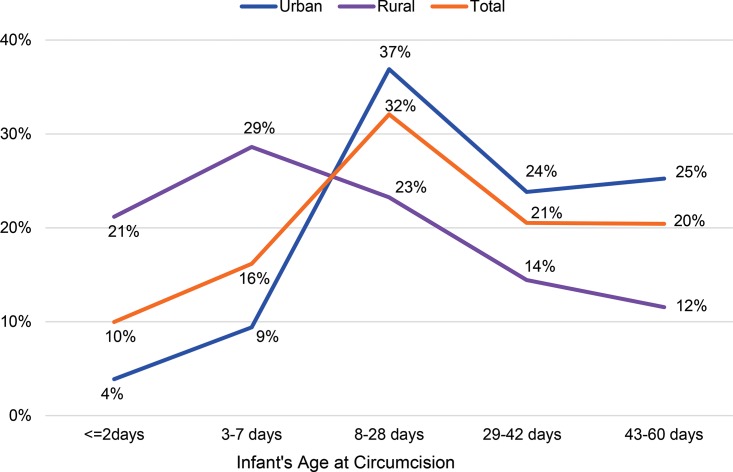
Percentage Distributions of EIMCs by Infant’s Age at Circumcision and Mother’s Residence Abbreviation: EIMC, early infant male circumcision.

As Figure 2 shows, nearly half of rural mothers who circumcised their infants did so within 7 days of birth (49.8% of infants, n = 362), whereas the rate of circumcision of infants among urban mothers was much higher after 7 days of birth (86.0% of infants, n = 1,151).

There were substantial differences between urban- and rural-dwelling mothers in terms of where their circumcised infant was delivered, who brought the infant for circumcision, and when and how often the infant was brought for follow-up (Table 3). Among mothers living in urban areas, attendance at the day 2 follow-up visit was close to universal (97.4%), whereas the rate among mothers living in rural areas was 84.6% (*P*<.001). The greatest difference, however, was seen in attendance at the day 7 follow-up visit; 49.9% among rural mothers compared with 82.2% among urban mothers (*P*<.001). On the day of circumcision, 77.5% of the infants were brought by mothers or female guardians alone (95% confidence interval [CI], 75.7% to 79.2%), and 22.5% were brought by both parents (95% CI, 20.7% to 24.3%). Rural-dwelling parents were more likely to bring the infant together (31.2% rural versus 17.7% urban parents, *P*<.001) (Table 3).

**FIGURE 3:**
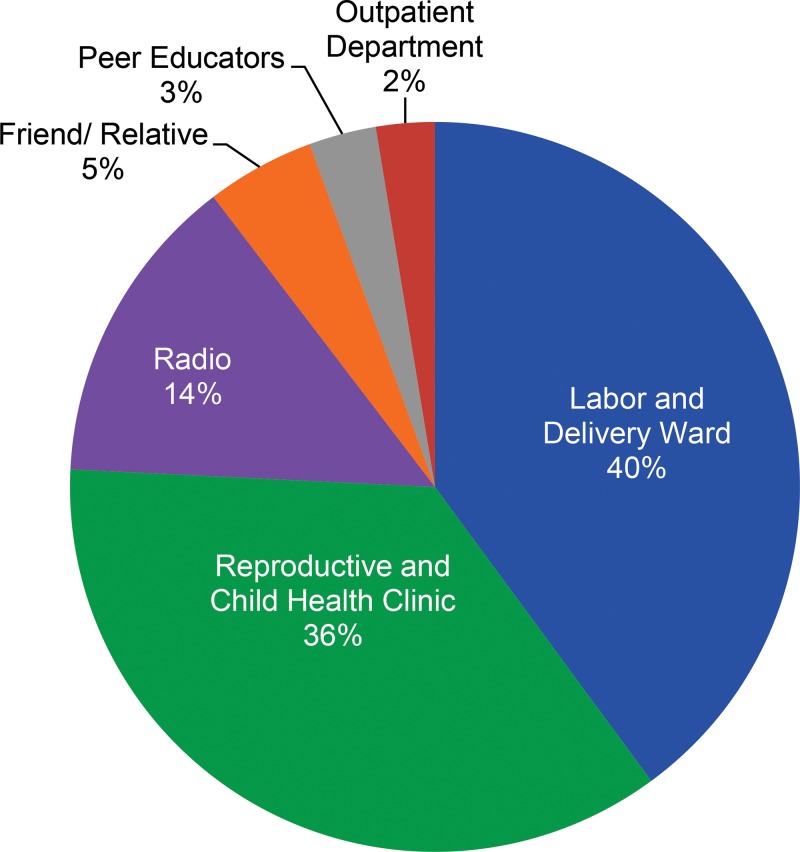
Where Parents/Guardians Heard About EIMC Abbreviation: EIMC, early infant male circumcision.

More than three-quarters (78%) of parents/guardians had heard of these services in a health facility setting, most often the labor and delivery ward (40%; range, 14%–44%) and the RCH clinic (36%; range, 35%–43%). Another 14% said they had heard about these services from the radio, 5% from a friend or relative, and 3% from a peer educator (Figure 3).

More than three-quarters of parents/guardians had heard of EIMC services in a health facility.

Six adverse events occurred among the 2,084 circumcisions conducted in the pilot project, for an overall AE rate of 0.4%. All AEs occurred during the circumcision procedure (intraoperatively). They included mild bleeding (n = 3), moderate bleeding (n = 1), and mild/excessive skin removal (n = 2). The AEs, which occurred in 2 of the 8 pilot facilities, occurred in procedures performed by assistant medical officers (2 AEs) and nurses (4 AEs). All AEs resolved completely. All providers performing EIMC in the pilot project had attended a competence-based 5-day training using a WHO Manual for EIMC.^8^ The EIMC providers in the pilot project included nurses (68%), physicians (18%), clinical officers (11%), and assistant medical officers (3%).

### Qualitative Study Findings

The quotes from themes presented in this paper address decision making. Other themes that emerged, including gender and satisfaction, will be presented in upcoming papers.

#### EIMC Information Received

In keeping with the program model, participants in the FGDs said that when the mother had been counseled on EIMC as part of her RCH services, she was the one who provided information about EIMC to her partner as part of the family decision-making process. This was heard from both the female and male FGD participants: Most male “acceptors” indicated that they had heard about EIMC from their wives.

*I got the [EIMC-related] information from my wife after she was advised by the doctors here*. —EIMC male acceptor, Iringa Referral Hospital

#### Decision Making

Although women were characterized as bringing information about EIMC into the home, more than 80% of women in both the acceptor and non-acceptor FGDs referred to the father as the decision maker about whether the child would be circumcised.

*It’s true that dads are the problem. If the man says the child won’t be circumcised till he turns 2 years, it will be like that no matter what. And I have no power to force it other way ‘round.* —EIMC female non-acceptor, Iringa Referral Hospital

More than 80% of women said the father was the decision maker about their infant’s circumcision.

Among female non-acceptors, more than 60% in FGDs indicated that they had wanted their son to be circumcised, but the father refused.

*I feel badly.… I wanted to circumcise him soon after birth, but his father refused because he said he is still young, wait until he grows up.* —EIMC female non-acceptor, Ipogolo Health Centre

Among some acceptors, decision makers involved the extended family, including the grandparents.

*First of all I talked to my husband. Secondly I talked with parents of both sides who are in-laws…. When they all agreed, then I made a decision to circumcise him.… Because this child has a grandfather and a grandmother. Everything should be shared with these elders.* —EIMC female acceptor, Iringa Referral Hospital

#### Misconceptions and Relation to Traditional Circumcision

FGD participants, particularly male non-acceptors, described 3 notable misconceptions. These were: (1) fear of suturing and resulting injury to the penis (a misconception since the EIMC procedure does not include suturing), (2) fear that the foreskin would be taken for witchcraft or another purpose, and (3) fear that the penis would not grow properly without the foreskin. Some male non-acceptors also equated the EIMC procedure with traditional circumcision, in which, typically, no pain medication is given.

*Not knowing how an infant is circumcised, as far as I know it, circumcision is done by operation. Now, stitching is where my fears lie, taking into account the delicacy of the infant’s skin … if the skin is to be stitched, I can’t imagine the type of stitches that are used!* —EIMC male non-acceptor, Iringa Referral Hospital

*They believe that the things that are cut might be taken to people who know how to use them … Maybe some people who have superstitious beliefs might have been asked to bring the child’s foreskin.* —EIMC female acceptor, Ipogolo Health Centre

*He does not want his son to be circumcised while he is still young, because if you do so his reproductive organs won’t grow at all.* —EIMC female non-acceptor, Iringa Referral Hospital

For some fathers who volunteered the information that they had undergone traditional circumcision, the recollections of their experience made them decide against their sons’ circumcision. None of these fathers were aware that EIMC is performed using devices and with pain medication.

*I was circumcised traditionally in the bush without anesthesia, without anything, you see? … Nowadays one is circumcised by using scissors, unlike in old days where a knife or something else was used … If you recall the way you were circumcised and think of your child going through the same process, you think, “Let him grow a bit.”* —EIMC male non-acceptor, Ipogolo Health Centre

*It is true that I opposed this circumcision … The reason for me to have such an argument is that I myself was circumcised [traditionally] when I was about 15 years old. … I can remember that there was a kid of about 5 years in the group. … In my observation he suffered, and I reached the conclusion that my parents had done the right thing when they decided that I should be circumcised [at an older age].* —EIMC male non-acceptor, Ipogolo Health Centre

## DISCUSSION

In the pilot project in Iringa region, 16.4% (2,084) of all male infants delivered in the 8 health facilities were circumcised. In larger hospitals, coverage tended to be lower (4%–8% of male infants delivered in the facility), although greater numbers of infants were circumcised because of the larger patient population. In facilities with relatively low delivery volume, there was greater coverage—more than 25%. It is likely that human resource constraints limited the number of circumcisions performed per day at facilities with a high volume of deliveries.

Human resource constraints may have limited the number of circumcisions performed per day at facilities with a high volume of deliveries.

The majority of the EIMCs were performed in the WHO-recommended window between 0 and 60 days after birth,^8^ and most occurred after 7 days of birth. Two-thirds of the EIMCs took place in the same facility where the infant was delivered; relatively few (6.3%) were performed on infants born at home. The follow-up rate was very high for the 2-day follow-up visit (93%) and lower for the 7-day visit (71%), with substantial differences between urban and rural residence. A total of 7.6% of infants circumcised were born to mothers with HIV. Only 8% of mothers indicated that they had heard of EIMC services from a friend or relative (5%) or a peer educator (3%), implying that community awareness was quite low at the time of the pilot project.

Urban and rural residence played a role in the age of the infant at circumcision and the time of return for follow-up visits. EIMC generally occurred later for infants from urban areas than for those from rural areas (mean of 28 days for urban residents versus 17 days for rural residents, *P*<.001). Because there is a limited window for EIMC (24 hours to 60 days after birth), there may be benefits for parents to seek services earlier; if the procedure needs to be delayed due to health facility constraints or ineligibility that may resolve over time (e.g., fever during health assessment), there may still be an opportunity to seek EIMC before the 60-day time frame. Although attendance at follow-up visits was very high among both groups for the 48-hour visit, infants with mothers who reported urban residence were 3.8 times more likely to return for a second follow-up visit. This may be attributable to barriers to health care access faced by rural residents. The service delivery model designed for EIMC following the pilot project must address urban and rural differences in access to services. These differences may affect potential EIMC outreach and logistics needed to ensure good follow-up rates; there may be a need to use rural health outposts for EIMC follow-up among rural residents.

On average, infants of urban mothers were circumcised later than infants of rural mothers. They also were more likely to be brought for the second follow-up visit.

The antenatal, perinatal, and postnatal periods are times when the mother and infant have multiple points of access to the health system.^14^^,^^15^ These times provide opportunities for the parents to make decisions for the immediate and longer-term health needs of their children. The 2010 Tanzania Demographic and Health Survey found that 65% of women whose last live birth occurred in the preceding 5 years did not receive a postnatal check-up.^14^ This indicates a large gap in infants being seen in the postnatal period, which, in addition to missing key health information and services, means that an opportunity for EIMC counseling may be missed. In the Southern Highlands zone, where Iringa region is located, 48% of babies are delivered at home.^14^

EIMC services are highly integrated with RCH and maternity services, and EIMC procedures are performed by the same health care providers and in the same locations in the health facilities. Given this integration, our analysis included the timing of the EIMC procedure in order to inform program planners as to when, in the spectrum of maternal, newborn, and child health services, parents might be most open to having their infant circumcised. A South African study found that parents have distinct preferences regarding the age of circumcision for their infants—based on their perceptions of factors such as wound healing, pain, and caretaking—and that the majority of the mothers and fathers were willing to circumcise their infants during the first week after birth.^16^

Our findings revealed that, although rural parents were more likely to seek EIMC before the infant was 2 days old, the majority of parents (73%) brought their child for circumcision between 8 and 60 days, meaning that the parents brought them back to the facility rather than having the infant circumcised before discharge from delivery. Despite WHO’s recommendation that the infant stay in the health facility for 24 hours after birth,^17^ anecdotal evidence suggests that, because of crowding in health facilities, mothers and infants are often discharged prior to 24 hours, making the male infants ineligible for circumcision. Similarly, because Tanzanian facilities generally do not allow birth companions in the delivery room, fathers are often not present at the delivery of their sons. Because fathers play an important role in deciding whether to circumcise male infants, their absence at delivery may mean a missed opportunity for EIMC before the mothers and infants are discharged. Given these realities, the programmatic implications of encouraging parents to circumcise the infant before discharge from the facility in the immediate postpartum period should be investigated. Potential behavior change communication models include encouraging women to discuss EIMC with their partners when they are attending ANC, or to integrate EIMC counseling specifically into services for the prevention of mother-to-child transmission of HIV, which men are more likely to attend. Further research may be warranted to look at the timing of the EIMC messages and counseling within the spectrum of RCH services, to explore whether earlier messages provide more time for mothers-to-be to discuss EIMC with the father.

This analysis focused on characteristics of families and infants and EIMC decision making. Gains in cultural acceptance of VMMC have been documented in Iringa region. Accounts suggest that circumcision has become socially desirable for a variety of reasons.^10^^,^^14^ However, the qualitative findings of this study suggest that acceptance of VMMC may not have as much of a positive effect on acceptance of EIMC as might be hoped. The specific reasons that non-acceptors gave for declining EIMC (fears about the delicacy of the skin of the infant and fears about growth of the penis) were quite different from those noted as barriers to VMMC, which had more to do with abstinence and relationship status. In addition, the positive social pressure for adult men in Iringa to be circumcised, based on the perception that male circumcision increases their sexual desirability, does not apply to EIMC.^10^^,^^18^

The reasons that non-acceptors declined EIMC were quite different from those noted as barriers to VMMC.

Fathers were the least exposed to messages about EIMC from health care providers but held greater decision-making power and had the most misconceptions about EIMC. This finding parallels findings in Kenya, where fathers were reported to be the primary decision makers regarding EIMC in 66% of the couples interviewed.^18^ A study in Zimbabwe noted similar barriers. Fathers had strong decision-making powers, and men who had been traditionally circumcised often had negative perceptions of EIMC.^19^ The current study and the Zimbabwe study support our conclusion that, for effective EIMC scale-up, program planners must consider the household decision-making process and find ways to reach men with accurate information about EIMC.^11^

A very low proportion (<10%) of EIMCs were performed on infants who were born at home. Reaching male infants who are born at home with EIMC will eventually become an important—and programmatically challenging—goal because approximately half of Tanzanian women deliver their babies at home.^14^ We found that the majority of infants were circumcised in the 8- to 60-day period, making the integration of EIMC with RCH—particularly immunization services—critical to reaching male infants born at home.

### Limitations

The study had a few limitations. Qualitative findings are limited to the original 4 facilities, but the pilot project expanded to include 4 additional facilities during the course of the study, so the quantitative data for all 8 sites were included in this analysis. However, it should be noted that the communities served by the original 4 facilities have a sociodemographic profile that is similar to that of the 4 additional facilities, so we would expect similar responses. Our strata for analyzing age at circumcision were based on “best guess” estimates, which considered the immunization schedule and assumptions about convenience of travel with a newborn.

**Figure:**
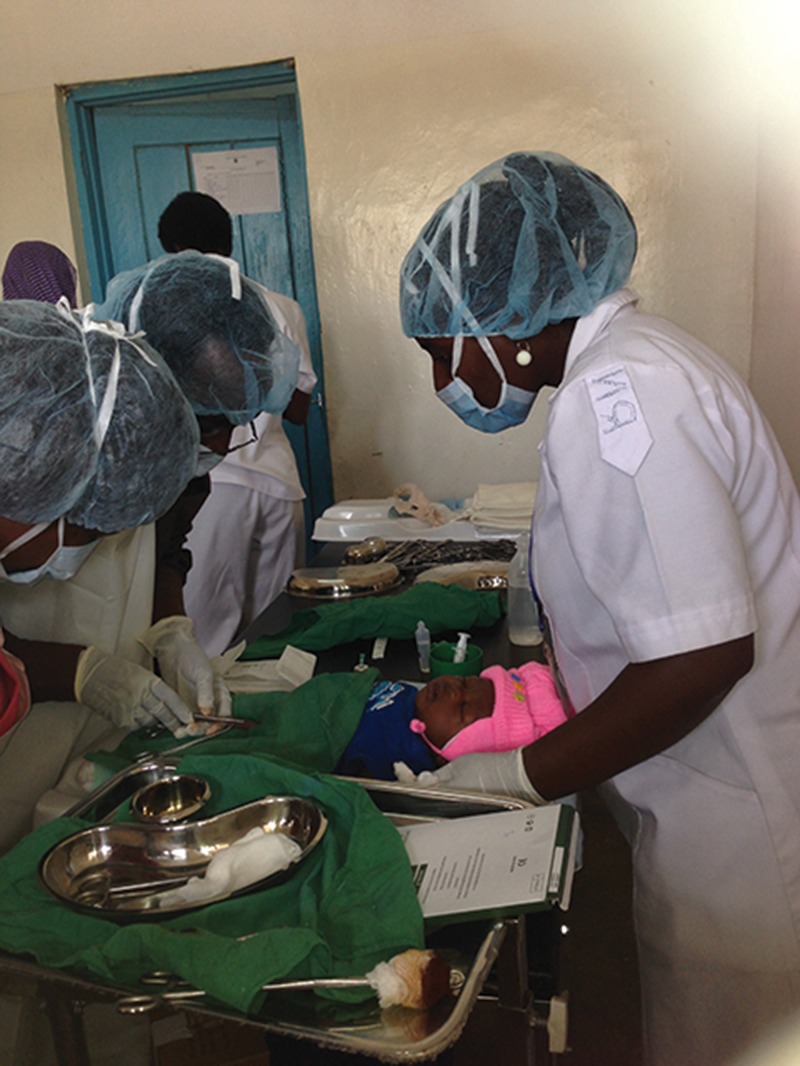
Providers in Iringa, Tanzania, perform early infant male circumcision using the Mogen clamp.

## CONCLUSION

EIMC services were introduced with a high level of safety in Iringa region, but it was noted that successes in scaling up VMMC did not translate into immediate acceptability of EIMC. Babies born at home comprised less than 7% of the infants circumcised in the pilot project. Care-seeking behavior associated with EIMC, including age of the infant at circumcision and time of return for follow-up visits, varied significantly between urban and rural dwellers. Strategies such as EIMC outreach activities and the use of rural health outposts for follow-up visits to overcome geographic barriers should be explored when planning to expand EIMC services.

Reaching male infants who are born at home will eventually become an important—and programmatically challenging—goal.

The majority of mothers heard about EIMC at a health facility rather than from radio messages or from community members. This highlights an important need and opportunity for expansion of community education and awareness-raising for EIMC via media outlets such as radio. It is particularly important that EIMC information and counseling reach men as well as women. Integrating EIMC counseling into services for prevention of mother-to-child transmission of HIV should be explored, because these services provide an opportunity for men and women to be counseled together. Women attending ANC should be encouraged to address the issue of EIMC with their partners and family early in their pregnancies. Based on our findings, targeted behavior change communication should be directed to fathers; using community forums to educate men about EIMC may be a good platform to explore.

## Notes

**Peer Reviewed**
